# Silver Ink Formulations for Sinter-free Printing of Conductive Films

**DOI:** 10.1038/srep20814

**Published:** 2016-02-09

**Authors:** Kate Black, Jetinder Singh, Danielle Mehta, Sarah Sung, Christopher. J. Sutcliffe, Paul. R. Chalker

**Affiliations:** 1 University Liverpool, Department of Engineering, Liverpool L69 3GH, Merseyside, England

## Abstract

Inkjet printing offers an attractive method for the deposition of metal interconnects in electronic systems and enables a low-cost, environmentally friendly route to manufacture. However, virtually all current metal inkjet processes require post-deposition sintering treatments to achieve the optimum electrical conductivity, because the growth mechanism involves coalescence of discrete nanoparticles. A manufacturing process that reduces the number of steps by directly printing silver, removing the need to sinter the printed metal, would be highly advantageous. Here we describe a, sinter-free process that results in the direct printing of crystalline silver. This process exploits the chemistries developed for Atomic Layer Deposition (ALD), to form the basis of a new ink formulation, which we term; Reactive Organometallic inks (ROM). These ROM ink formulations are capable of depositing low temperature, high conductivity metal films, without the need for subsequent sintering treatments. To reduce the temperature for direct formation of metallic Ag, we have added an alcohol as a catalytic reducing agent to dissociate the organometallic component. Silver films printed from our novel ROM ink, on a glass substrate at 120 °C, are electrically conductive with a typical resistivity as low as 39.2% that of bulk silver, without the need for sintering.

Inkjet printing has been investigated as an alternative production tool for the manufacture of conductive components, for use in electronic devices. This is due to the many benefits associated with inkjet printing, such as low-cost, additive and efficient handling of expensive materials. Commonly used inks for the inkjet production of conductive components are presently based on conducting polymers, nanoparticle (NP) solutions or dispersants^1^,^2^,^3^,^4^,^5^, all of which have associated drawbacks. Volkman *et al.*^3^ performed mechanistic studies on the sintering of silver nanoparticles. To overcome the problem of aggregation and flocculation, observed when using pure metal nanoparticle inks, a thiolate encapsulating surfactant was used to treat 3 nm silver particles to achieve sintered films at temperatures above 175 °C in air. Sintering is essential to obtain the conductivities required for electronic applications. For example, the effects of sintering on electrical performance and microstructure for an inkjet-printed copper nanoparticle ink were explored by Niittynen *et al.* They used laser and intense pulsed light (IPL) sintering in order to achieve films with conductivities of more than 20% that of bulk copper^4^. However sintering techniques can cause major drawbacks. In many cases subsequent sintering steps require high temperatures that are not compatible with the polymer substrates that are commonly employed in plastic electronics, such as polyethylene terephthalate or polycarbonate. Grouchko *et al.*^6^ recently overcame some of these issues by employing a room temperature, “built in” sintering mechanism that successfully produced Ag films of conductivities as high as 41% of that of bulk silver. To obtain these conductivity values, a chloride salt (e.g. NaCl) or HCl vapor was employed to strip a polymeric (polyacrylic acid sodium salt) electrosterically stabilizing coating from the ~15 nm diameter Ag NP feedstock. The sintering mechanism consists of spontaneous coalescence and Ostwald ripening, driven by the surface-to-volume energy of the very small nanoparticles. The size-dependence of the Ag NPs on these processes has recently been elucidated^7^. All of these NP-based processes inherently involve sintering processes, whether they are chemical, thermal, laser or UV activated. An alternative to the NP approach is to employ a chemical ink formulation, where the silver source is a molecular precursor or cation. Conductive inks that are based on a solution, rather than a suspension, have gained interest in the past couple of years^8^,^9^,^10^,^11^. One solution-based ink approach is the Metalorganic Decomposition (MOD) variety. For example, Jahn *et al.*^11^ investigated silver printing from an aqueous transition metal complex [AgO_2_C(CH_2_OCH_2_)_3_H] based MOD ink. They achieved metallic silver features with conductivities as high as 2.7 × 10^7^ Sm^−1^, which corresponds to a conductivity that is 43% that of bulk silver, although a sintering temperature of 250 °C was required to obtain such conductivities. MOD inks obviate some issues associated with NP ink, i.e. nozzle clogging, however numerous printing passes are required to obtain an adequate sheet resistance. Post-treatment sintering processes are also still required to fully consolidate the conductive films, if the initial growth process is from discrete NP intermediates which is common in MOD ink processes. To target a layer-by-layer silver film growth mechanism, an alternative approach has been to employ MOD inks in a sequential Reactive Inkjet (RIJ) process. This approach involves an initial printed MOD ink step, followed by a secondary printed reducing ink step (or vice versa). The chemical reaction between these reagents synthesizes the elemental metal directly on the surface^12^,^13^,^14^.

In this paper, we seek to advance the RIJ strategy by combining the dual metal organic ink and the reducing ink into a single ink formulation. The focus of this paper has been to minimise the number of printing steps and remove the sintering step. We have developed a new class of ink with which we exploit surface chemistries to achieve a sinter-free route to printing metal tracks. Specifically this involves the careful tailoring of the substrate temperature and the enthalpy of reaction of the organometallic (OM) component with a co-reducing agent, in this instance an alcohol, to ensure a surface-driven process rather than a liquid-phase homogeneous process. Through this approach, the OM reduction rate can be driven to directly print crystalline films, layer-by-layer, so that sintering is not necessary to make them electrically conductive. We have coined the term Reactive Organometallic (ROM) inks for this class of solution-based inks to distinguish the process from the MOD and RIJ mechanisms. These ROM inks promise to provide a flexible, lower thermal budget and faster route for the manufacture of printed electronics.

A range of organometallic precursors are potentially suitable for the ROM methodology because of their solubility in ink carrier solvents and reactivity at low temperatures. For example, the silver hexafluoroacetylacetonate cyclooctadiene [(hfac)(1,5-COD)Ag] precursor has been previously investigated as a silver source for the metal organic chemical vapor deposition (MOCVD) of silver on titanium nitride adhesion layers^15^. It was reported that silver films could not be obtained; however, when the neutral COD ligand was replaced with vinyltriethylsilane (VTES); it was found that Ag films could be deposited at a substrate temperature as low as 180 °C. Subsequently, Bahlawane *et al.*^16^ demonstrated the growth of silver films by chemical vapor deposition using [(hfac)(1,5-COD)Ag] and various alcohols. The process exploits the catalytic reactivity of cationic silver, and the reactivity of alcohols with silver surfaces. The driving force in this process was attributed to the catalytic oxidative-dehydrogenation of alcohols. The same precursor has also been used in Atomic Layer Deposition (ALD) to grow silver films in supercritical CO_2_ using H_2_ and acetone as the reducing agents^17^. In the ALD process, the role of the propanol was to reduce the [(hfac)(1,5-COD)Ag] to metallic silver. The role of the alcohol in catalysing the process is significant and it has been reported that the affinity of cationic silver to alcohols and aldehydes leads to its chemical reduction^18^. Herein, this approach has been modified to prepare a reactive organometallic-based ink formulation suitable for inkjet processing to achieve sinter-free printing of silver films with high electrical conductivity, whilst being deposited at a low thermal budget.

## Results and Discussion

**Figure 1:**
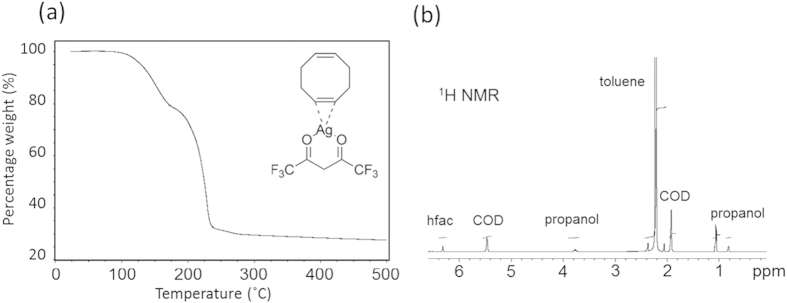
(**a**) Thermogravimetric analysis of the decomposition of the pure [(hfac)(1,5-COD)Ag]ink component (b) 1 H NMR of the complete [(hfac)(1,5-COD)Ag]ROM ink formulation.

**Figure 2:**
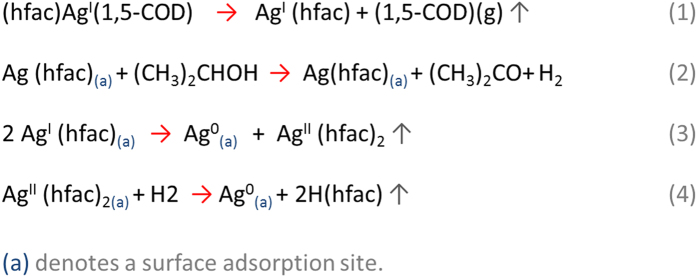
Equation 1 shows the onset of decomposition via the loss of the 1,5-cycloocatadiene (COD) ligand and equations 2–4 shows the subsequent reduction of the Ag metal.

To understand the thermal decomposition of the pure OM component of the ink, [(hfac)(1,5-COD)Ag] (molar mass = 423.1 g/mol), thermogravimetric analysis was performed under a nitrogen ambient atmosphere, as shown in Fig. 1a. The onset of decomposition is indicated from 110 °C via the loss of the 1,5-cycloocatadiene (COD) ligand, as shown in Fig. 2, equation one. This temperature coincides with the melting of the crystalline solid. Above approximately 150 °C the decomposition of the Ag(hfac) component occurs showing weight-loss of the fugitive hfac ligand, but the reduction is not complete until 220 °C leaving a residual weight of 27.7%. This closely matches the formation of metallic silver (molar mass = 107.87 g/mol) which would leave a theoretical residue of 25.5% if perfectly pure. To reduce the temperature of metallic Ag formation from 220 °C, we have added an alcohol as a catalytic reducing agent to dissociate the OM in the ink formulations presented here. We explored the printing of silver films via a range of ink formulations with varying catalytic alcohol concentrations. A series of inks were formulated by dissolving [(hfac)(1,5-COD)Ag] in a toluene carrier. Varying proportions of propan-2-ol were added to the solution to promote the elimination of the hfac and reduce the incorporation of unwanted impurities from the ligand.

^1^H NMR was used to confirm the concentration of the silver metal organic component and alcohol in a solution of the toluene carrier; ^1^H NMR: (400 MHz, C_6_D_6_) δ 6.8–7.4 (m, 5H,CH, C_7_H_8_) 6.3 (s,^1^H,F_3_CC(O)CHC(O)CF_3_) 5.5 (s, 4H,CH, 1,5-cyclooctadiene) 3.7 (m,^1^H,HOCH(CH_3_)_2_) 2.0–2.4 (t,3H,CH_3_, C_7_H_8_) 1.9 (d,8H,CH_2_, 1,5-cyclooctadiene) 1.1 (d,6H,HOCH(CH_3_)_2_). Figure 1b shows the ^1^H NMR spectrum of this ink.

**Figure 3:**
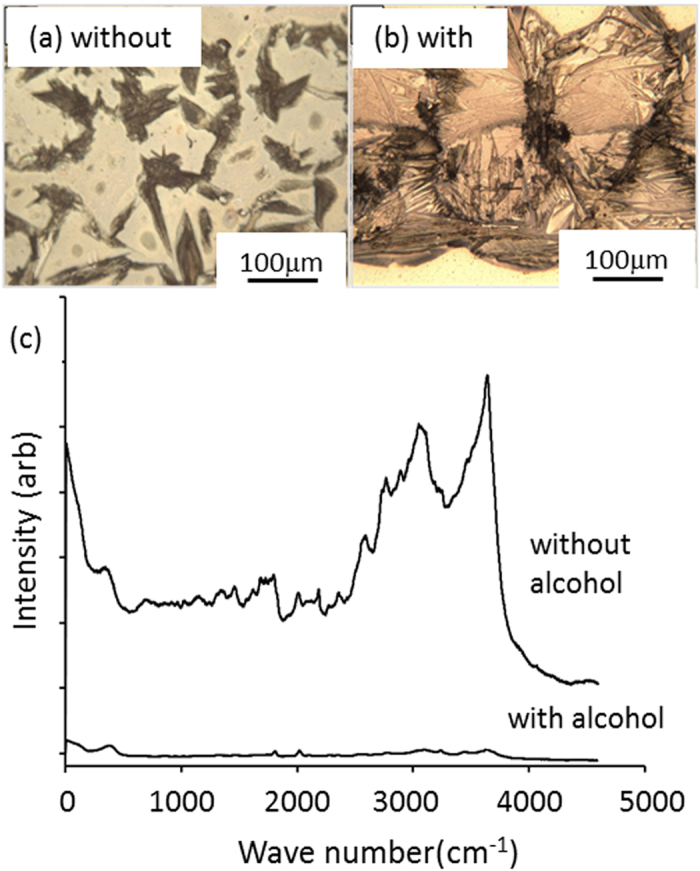
Optical micrographs of printed materials (**a**) with-out and (**b**) with excess alcohol. (**c**) Raman spectra of printed deposits with and without alcohol addition.

The effect of substrate temperature was explored using the ink formulation 0.1 M:0.2 M (organometallic:alcohol) over the temperature range of 90 °C to 120 °C. The range was chosen to investigate: (i) the temperature sensitivity on the contribution of the propan-2-ol in removing the hfac ligand, and (ii) the consequences for the purity of the printed silver. The effect of temperature is critical in determining the possible application of printing in situations where the thermal budget during manufacture has to match the stability of the substrate material (e.g., plastic or photovoltaic PV cell material, etc.). Table 1 shows the effect of substrate temperature on the electrical resistivity of Ag films printed at 90 °C to 120 °C. For an ink composition of 0.1 M hfac and an excess of alcohol, it can be seen that the resistivity is a sensitive function of the substrate temperature. In the narrow band of 90 °C–120 °C, the resistivity reduces by a factor of more than 100. We interpret this to be the result of a change in purity, microstructure and the direct growth habit of silver, in the presence of the catalytic alcohol reductant, during the evaporation of the toluene carrier. The pivotal role of the alcohol addition is shown in Fig. 3a,b, which shows optical micrographs of silver printed from 0.1 M ink, with and without alcohol, using the same printing conditions.

Without any alcohol (Fig. 3a), the deposit is a disconnected residue. With the alcohol (Fig. 3b) a crystalline track of silver was formed from each droplet, as-printed in a stepwise manner, containing dense interlocking crystals. X-ray diffraction spectroscopy (XRD) (Fig. S3) shows that the films printed with catalytic alcohol are pure silver. To further evaluate the composition of the printed materials, Raman spectra were also measured using the green line (λ= 514 nm) of an Ar+ ion laser. The intense Raman spectrum recorded from the film deposited without alcohol (Fig. 3c) is a complex series of bands attributed to hydrocarbon and C-F bonded organic residues. The spectrum from a sample printed with alcohol (Fig. 3c) has a much lower relative intensity confirming that nearly pure metal has been printed with the remaining noise probably associated with adsorbates on the surface of the metallic silver track.

**Figure 4:**
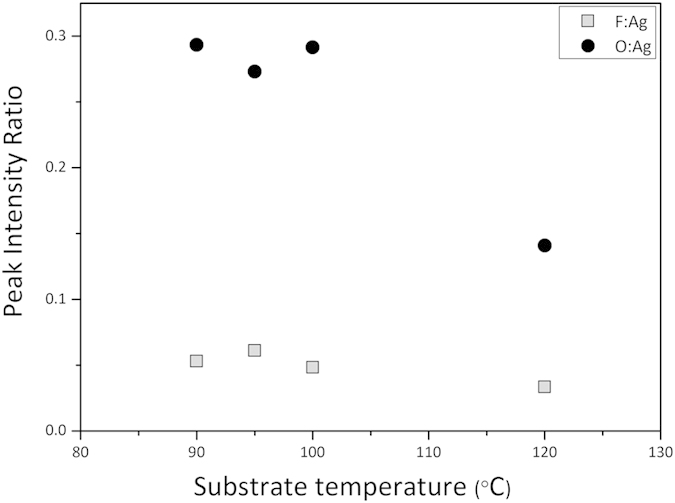
Elemental ratios of O:Ag (•)and F:Ag (◻) XPS intensities within the silver films printed between 90 °C–120 °C.

To understand the reason for the marked change in resistivity (Table 1), the films were examined with X-ray photoelectron spectroscopy (XPS) and scanning electron microscopy (SEM). The XPS measurements were recorded with Al kα radiation and estimates of the constituent elements were made from the peak areas of the F(1s), O(1s) and Ag(3d) intensities (see supplementary information Figure S2). The decrease in the F:Ag and O:Ag ratios demarks the onset of the catalytic reduction process. Prior to XPS analysis, the surface of the samples was sputtered with Ar ion bombardment to remove any adventitious surface contamination and reveal the representative sub-surface composition. To effectively compare the oxygen and fluorine incorporation within the samples printed at different temperatures, the ratio of O:Ag and F:Ag XPS intensities was used (Fig. 4). Both ratios show a decrease of contamination for the silver deposited at 120 °C. This is consistent with the Raman data and suggests that a transition to metal deposition is achieved at a threshold substrate temperature between 100 and 120 °C. We conclude that the metallic reduction takes place by chemical reactions 2–4 displayed in Fig. 2, whereby (a) denotes a surface adsorption site. The hydrogen that is liberated from the alcohol during the formation of the aldehyde, (Fig. 2 eq. 2) promotes the reduction of the disproportionation Ag II (hfac)_2_ product (Fig. 2 eq. 3) into the metallic silver film (Ag^0^) and volatile H(hfac) leaving groups (Fig. 2 eq. 4). Early high-resolution electron energy loss spectroscopy mechanistic studies^18^ on the chemical vapor deposition of copper from the [Cu(hfac)(COD)] analogue, pointed to the feasibility of the adsorbate intermediates outlined in reactions 3 and 4 in Fig. 2. In the absence of the alcohol, the (hfac) ligands cannot be removed from the surface to enable the Ag(hfac)_2_ dimer to evaporate if temperatures are lower than ~110 °C. At low temperature, without alcohol, more fluorine and oxygen is incorporated into the printed Ag films.

**Figure 5:**
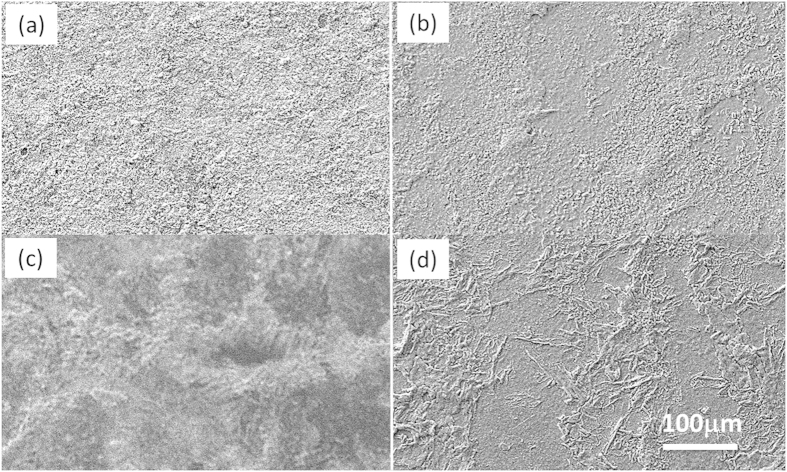
SEM images showing the microstructure of Ag films deposited at 120 °C using 0.1 M [(hfac)(1,5-COD)Ag]ink with alcohol molarities of (**a**) 0.00 M, (**b**) 0.05 M, (**c**) 0.20 M and (**d**) 0.10 M.

Figure 5 shows the influence of the propan-2-ol content in the 0.1 M [(hfac)(1,5-COD)Ag] ink on the resulting microstructure of the films. At a printing temperature of 120 °C, and for ten passes of the print head, adding alcohol evidently influences the nucleation and growth process. Without alcohol, the film consists of small granular features with some open surface porosity. With the addition of 0.05 M of alcohol the microstructure of the silver develops larger terraces of a relatively smooth film. For the 0.1 M:0.1 M ratio of organometallic to alcohol the transition to flatter areas of silver occurs with a coverage of extended ridge structures; this microstructure coincides with the lowest resistivity values. However, if the alcohol added is increased to “excess” (e.g. 0.2 M) the surface of the deposited silver becomes much rougher once again. From this it is concluded that there is an optimum molar precursor to alcohol ratio that is close to one-to-one.

**Figure 6:**
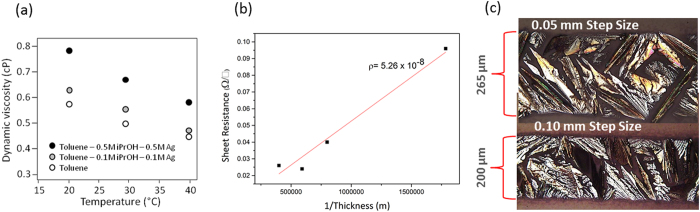
(**a**) Dynamic viscosity of the [(hfac)(1,5-COD)Ag]based ink formulations as a function of temperature. (**b**) Influence of number of print passes on the resistivity. (**c**) Ag tracks printed at 10 mm/s with a 0.05 mm and 0.10 mm step size, using the 0.5 M:0.5 M ink formulation.

The dynamic viscosity of the ink formulations was also examined as a function of the alcohol molarity and the concentration of the [(hfac)(1,5-COD)Ag] component as shown in Fig. 6(a). The effect of increasing both the concentrations of alcohol and silver precursor is to increase the overall dynamic viscosity. This change of fluid properties is important as it has ramifications on the printability of the ink such as step-size and print speed conditions under which silver can be deposited. The inkjet printing process can use multiple passes to build up the thickness of printed features and the effect of this on the films resistivity is shown in Fig. 6(b). From the gradient of the plot we observe an average resistivity of 5.26 × 10^−8^ Ω.m from films of four different thicknesses (0.5 to 2.2 μm). Films printed with 15 passes give the lowest resistivity value of 4.1 × 10^−8^ Ω.m (thickness 1.6 μm) which is 39.2% that of the resistivity of bulk silver. Figure 6(C) shows optical micrographs of silver tracks printed with the ink 0.5 M:0.5 M (organometallic: alcohol) formulation at 120 °C and with a print head speed of 10 mm/s. The target track width was 200 μm, however at a step size of 0.05 mm, the track printed extended out to a width of up to 265 μm with poorly delineated side walls. By doubling the step-size to 0.1 mm the printed track then conformed to the target width and the edge definition was dramatically improved.

Table 2 shows a comparison of the electrical resistivities of silver films printed using a range of alternative ink types and approaches. The metallorganic decomposition (MOD) inks reported, all employ a dual source approach that involves the sequential jetting of co-reagents followed by thermal or chemical sintering mechanisms. These are compared to two Ag NP-based ink processes that also exploit thermal or chemical treatments to sinter the film microstructure. The novel Ag ROM ink methodology presented here, produces a thermally activated catalytic reaction to directly reduce the silver OM precursor as the toluene carrier evaporates. All of the processes detailed in Table 2 produce conductive silver films with resistivities in the range of 2 × 10^−8^ to 2.63 × 10^−7^ Ω.m. Our ROM ink involves an intrinsically different approach to all other studies since we directly produce crystalline silver films at a substrate temperature of 120 °C. Multiple print passes are required to build the film thickness, but only a single source (ink) is required, and sintering is unnecessary since we have directly printed crystalline silver.

## Conclusions

This article reports the characterisation and application of novel Reactive Organometallic ink formulations for the sinter-free printing of high conductivity crystalline silver films. The addition of an alcohol, in this case propan-2-ol, is shown to be catalytic in the removal of the hexafluoroacetylacetonate ligands from the [(hfac)(1,5-COD)Ag] silver precursor. Sinter-free deposition of conductive silver films is achievable at a printing temperature as low as 110 °C. Below this temperature removal of the hfac ligand becomes incomplete, which subsequently degrades the electrical properties of the silver films. The 0.5 M:0.5 M organometallic-alcohol ROM ink formulation allows for the printing of crystalline silver films that exhibit electrical resistivities as low as 4.1 × 10^−8^ Ω.m which is 39.2% that of the resistivity of bulk silver (1.59 × 10^−8^ Ω.m).

## Methods

### Materials and equipment

The materials silver hexafluoroacetylacetonate cyclooctadiene [(hfac)(1,5-COD)Ag] and propan-2-ol were used, as supplied, by Sigma Aldrich with no further purification. Anhydrous toluene was supplied by Romil and further passed through a solvent drying system prior to use. Varying concentrations of [(hfac)(1,5-COD)Ag] were dissolved in anhydrous toluene in a glove box. This was followed by the addition, in a volumetric flask, of propan-2-ol to form a series of organometallic:alcohol ink ratios (0.1:0.1 M to ‘excess’ alcohol). The volumetric flask was then filled with toluene.

### Print pattern

All silver inks were inkjet-printed using a Microfab Jetlab x4 printing systems (Microdrop Technologies GmbH, Germany) in ambient atmosphere. All films were removed from the heated stage immediately after printing. One print pass took ~2 minutes per pass for a 1 cm^2^ area. The print head used was a MD-K-140 dispenser with a 50 μm diameter nozzle. The ROM silver inks were printed at 30 V, 36 μs pulse width, a frequency of 300 Hz and drop spacing between 0.05 to 0.1 mm. A typical waveform for the printing process is shown in Figure S1.

### Characterization

Crystallographic characterisation of the printed silver layers was performed using X-ray diffraction (XRD) and Raman spectroscopy. X-ray diffraction was performed on a Rigaku MiniFlex diffractometer using Cu Kα radiation (λ = 0.154051 nm, 40 kV, 50 mA) spanning a 2θ range of 10° to 60° at a scan rate of 0.01°/min. Raman spectra were obtained with a Jobin-Yvon LabRam HR consisting of a confocal microscope coupled to a single grating spectrometer. Raman measurements were performed using a confocal aperture of 300 μm to limit the light and so improve the quality of signal. The measurements were spanned with 0 to 4,500/cm of four accumulations, and the exposure time was 10 s. All of the spectra were observed using an incident wavelength of 325 nm from a He-Cd laser. To determine the electrical characteristics of the printed Ag samples, 4-point probe measurements were performed at room temperature using an Advanced Instrument technology CMT – SR2000N. SEM images where taken with a JEOL 6300F FE-SEM with an accelerating voltage of 20 kV. Optical images where taken using a Leitz Wetzlar Metalloplan microscope in transmission and reflection mode.

## Additional Information

**How to cite this article**: Black, K. *et al.* Silver Ink Formulations for Sinter-free Printing of Conductive Films. *Sci. Rep.* **6**, 20814; doi: 10.1038/srep20814 (2016).

## Supplementary Material

Supplementary Information

## Figures and Tables

**Table 1:** Electrical resistivity from Ag films printed on glass between 90 °C and 120 °C, from a 0.1 M [Ag(hfac)(COD)] ink with excess alcohol.

Substrate temperature	90 °C	95 °C	110 °C	120 °C
Resistivity (Ω.m)	1.89 × 10^−5^	1.88 × 10^−5^	5.26 × 10^−7^	1.68 × 10^−7^

**Table 2:** Literature survey and the results of this study highlighting comparative conditions for the inkjet printing of conductive silver pattern using various nanoparticle and MOD inks.

Type of ink	Sintering temperature ( °C)	Other post processing steps	Resistivity (Ω.m)	Reference
Ag dual source MOD	150 °C 60 min	Dried RT 10 min	2–3 × 10^−8^	12
Ag dual source MOD	150 °C 60 min	Washed with ammonia	5.4 × 10^−8^	13
Ag dual source MOD	RT	Submerged in hydroquinone	1.54×10^−7^	14
Ag nanoparticle	RT	HCl vapor/ NaCl	3.8 × 10^−8^	6
Ag nanoplates	100 °C 15 min	–	2.63 × 10^−7^	19
Ag ROM (this work)	N/A	N/A	4.06 × 10^−8^	–
