# Supplementary material for: Silver Ink Formulations for Sinter-free Printing of Conductive Films
Source: Sci Rep. 2016 Feb 9;6:20814. doi: 10.1038/srep20814 (PMC4809139; doi:10.1038/srep20814)
Supplement: Supplementary Information [file srep20814-s1.pdf]

Supplementary information
Silver Ink Formulations for Sinter-free Printing of Conductive Films

Kate Black*, Jetinder Singh, Danielle Mehta, Sarah Sung, Christopher. J. Sutcliffe and Paul. R. Chalker

1. Preparation of example silver ink formulations

1.1. *0.1M Ag(hfac)(COD), 0.1M 2-propanol in anhydrous toluene.*

Materials Ag(hfac)(COD) and 2- propanol were used as supplied by Sigma Aldrich with no further purification. Anhydrous toluene was supplied by Romil and further passed through a solvent drying system, prior to use. In a glove box, silver hexafluoroacetylacetonate cyclooctadiene (Ag(hfac)(COD)) (1.1g, 2.5mmol), was charged to a 25ml volumetric flask, followed by 10ml of anhydrous toluene. 2-propanol (190µL, 2.5mmol) was subsequently added to the Ag(hfac)(COD) solution. The volumetric flask was then filled with toluene. Concentration of the solution was confirmed by ^{1}H NMR.
^{1}H NMR: (400MHz, C_6D_6) δ 6.8-7.4 (m, 5H,CH, C_7H_8) 6.3 (s,1H,$F_3CC(O)CHC(O)CF_3$) 5.5 (s, 4H,CH, 1,5-cyclooctadiene) 3.7 (m,1H,$HOCH(CH_3)_2$) 2.0-2.4 (t,3H,CH_3, C_7H_8) 1.9 (d,8H,CH_2, 1,5-cyclooctadiene) 1.1 (d,6H,$HOCH(CH_3)_2$).

1.2. *0.1M Ag(hfac)(COD), 0.2M 2-propanol in anhydrous toluene.*

Materials Ag(hfac)(COD) and 2- propanol were used as supplied by Sigma Aldrich with no further purification. Anhydrous toluene was supplied by Romil and further passed through a solvent drying system, prior to use. In a glove box, silver hexafluoroacetylacetonate cyclooctadiene (Ag(hfac)(COD)) (1.1g, 2.5mmol), was charged to a 25ml volumetric flask, followed by 10ml of anhydrous toluene. 2-propanol (390µL, 5mmol) was subsequently added to the Ag(hfac)(COD) solution. The volumetric flask was then filled with toluene. Concentration of the solution was confirmed by ^{1}H NMR.
^{1}H NMR: (400MHz, C_6D_6) δ 6.8-7.4 (m, 5H,CH, C_7H_8) 6.3 (s,1H, $F_3CC(O)CHC(O)CF_3$) 5.5 (s, 4H,CH, 1,5-cyclooctadiene) 3.7 (m,1H,$HOCH(CH_3)_2$) 2.0-2.4 (t,3H,CH_3, C_7H_8) 1.9 (d, 8H, CH_2, 1,5-cyclooctadiene) 1.1 (d, 6H,$HOCH(CH_3)_2$)

1.3. *0.5M Ag(hfac)(COD), 0.5M 2-propanol in anhydrous toluene.*

Materials Ag(hfac)(COD) and 2- propanol used as supplied by Sigma Aldrich with no further purification. Anhydrous toluene was supplied by Romil and further passed through a solvent drying system, prior to use. In a glove box silver hexafluoroacetylacetonate cyclooctadiene (Ag(hfac)(COD)) (5.3g, 12.5mmol), was charged to a 25ml volumetric flask, followed by 10ml of anhydrous toluene. 2-propanol (950µL, 12.5mmol) was subsequently added to the Ag(hfac)(COD) solution. The volumetric flask was then filled with toluene. The solution was filtered. Concentration of the solution was confirmed by ^{1}H NMR. ^{1}H NMR: (400MHz, C6D6) δ 6.8-7.4 (m, 5H,CH, C_7H_8) 6.3 (s, 1H, $F_3CC(O)CHC(O)CF_3$) 5.5 (s, 4H,CH, 1,5-cyclooctadiene) 3.7 (m, 1H, $HOCH(CH_3)_2$) 2.0-2.4 (t,3H,CH_3, C_7H_8) 1.9 (d, 8H, CH_2, 1,5-cyclooctadiene) 1.1 (d,6H,$HOCH(CH_3)_2$)

2. Wave forms employed for Ag ROM-ink printing

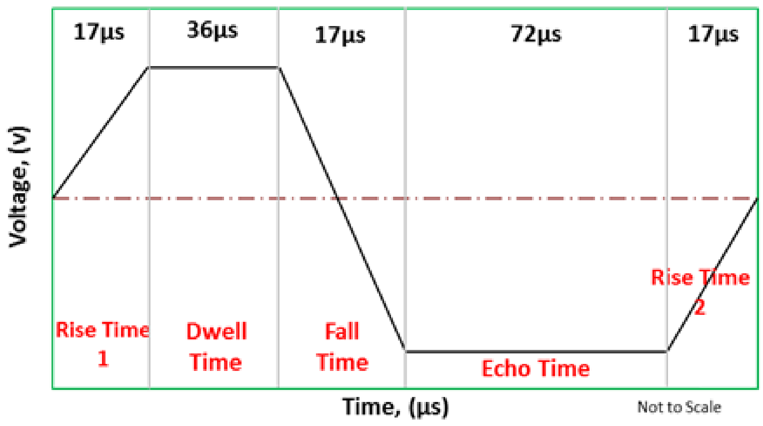

Figure S1. A. Waveform used for all Ag ROM ink experiments.

3. Depth profiles of printed film constituents measured by X-ray Photoelectron Spectroscopy

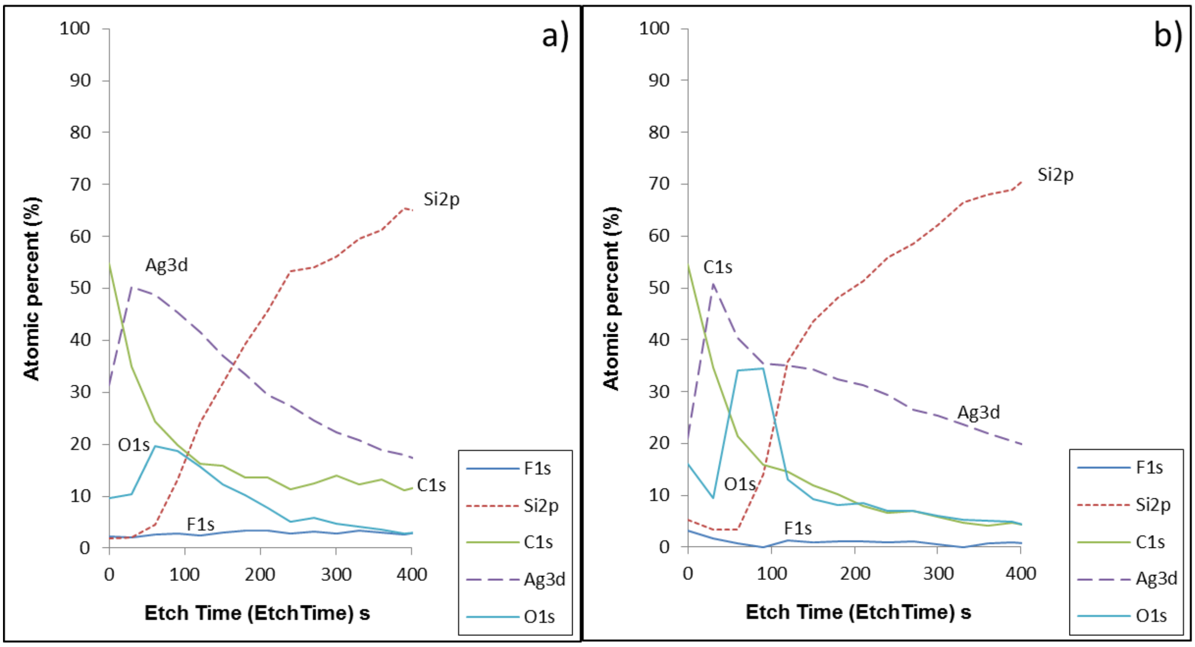

Figure S2. XPS depth profiles of the F(1s), Si(2p), C(1s), Ag(3d) and O(1s) intensities measured during argon ion sputtering of silver films printed at a) 90°C and b) 120°C.

The XPS measurements were recorded with Al-kα radiation and estimates of the constituent elements were estimated from the peak areas of the F(1s), Si(2p), C(1s), Ag(3d) and O(1s) intensities.

4. X-ray diffraction patterns of the ROM ink printed Ag films

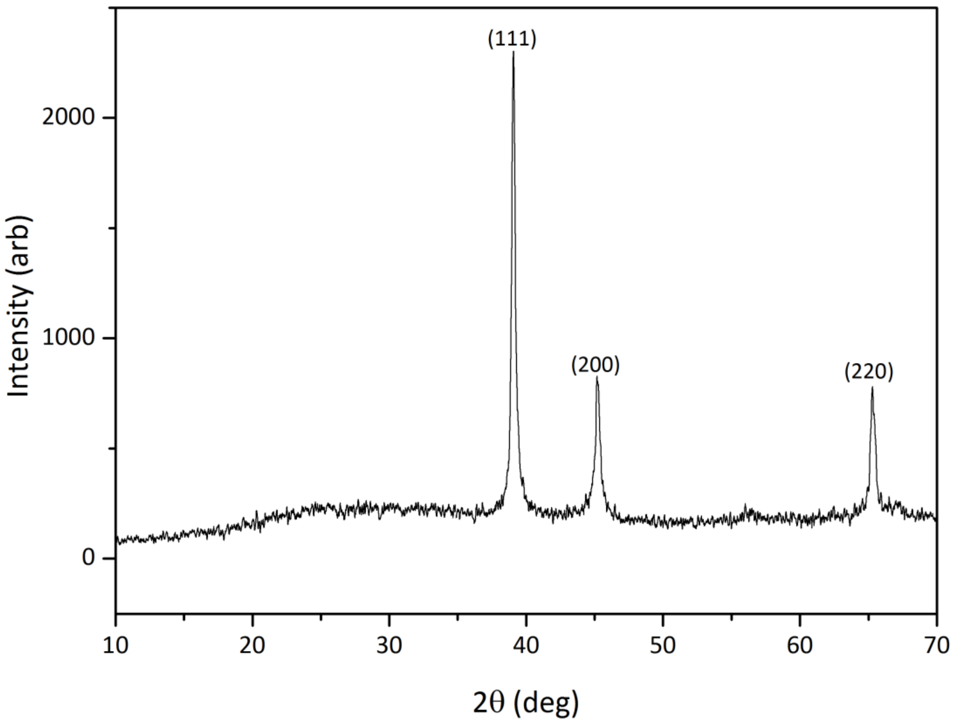

Figure S3. X-ray diffraction spectroscopy of the new Ag reactive organometallic (ROM) ink on glass substrates at temperatures of 120˚C revealing no phases present apart from pure metallic silver.
